# Effect of Different Genotypes and Harvest Times of Sage (*Salvia* spp. Labiatae) on Lipid Oxidation of Cooked Meat

**DOI:** 10.3390/antiox12030616

**Published:** 2023-03-02

**Authors:** Kathrine H. Bak, Susanne Bauer, Friedrich Bauer

**Affiliations:** Institute of Food Safety, Food Technology and Veterinary Public Health, University of Veterinary Medicine Vienna, Veterinärplatz 1, 1210 Vienna, Austria

**Keywords:** antioxidant capacity, genotype, TBARS, peroxide value, sage, superoxide anion scavenging activity

## Abstract

Lipid oxidation is the primary non-microbial reason for quality deterioration of meat and meat products. Lipid oxidation can be prevented or delayed by antioxidants. In this study, 15 sage (*Salvia* spp. Labiatae) extracts (five genotypes, three harvest times) were tested for their ability to reduce lipid oxidation (peroxide value (PV) and thiobarbituric acid reactive substances (TBARS)) in ground, uncured, cooked porcine and bovine meat (60%/40% mixture) during 14 days of refrigerated storage. Additionally, total phenolic content was determined, and the antioxidant capacity of the extracts was measured as radical scavenging activity (2,2-diphenyl-1-picrylhydrazyl assay), reducing power, and superoxide anion scavenging activity. All 15 sage extracts were able to reduce lipid oxidation, though showing expected differences depending on genotype and harvest time. The extracts of *S. officinalis* accession from Foggia, Italy performed better than the other genotypes when looking at the entire storage period and considering both PV and TBARS. Of the applied methods for determining antioxidant capacity, superoxide anion scavenging activity proved to be the best determinant of the ability of sage to reduce lipid oxidation in the meat sample.

## 1. Introduction

It is well-known that lipid oxidation is the primary non-microbial reason for quality deterioration in meat and meat products [1,2,3,4]. Lipid oxidation affects not only flavor, but also color, texture, nutritional value, and food safety [2,3]. Formation of secondary lipid oxidation products (carbonyls, hydrocarbons, alcohols, furans) is known to lead to off-flavors in foods [1,5]. Secondary lipid oxidation products include aldehydes such as pentanal, hexanal, 4-hydroxynonenal, and malondialdehyde (MDA) [6].

Antioxidants are able to prevent or delay oxidation even though they are present in low concentrations compared to the oxidizable substrate [7]. Antioxidants can be grouped according to their mode(s) of action. Chain-breaking (primary) antioxidants, which intercept free radicals generated during lipid oxidation, are generally the most efficient group of antioxidants [7,8]. Secondary antioxidants work by suppressing oxidation initiators or accelerators (e.g., by chelation of prooxidative metals) or by regenerating primary antioxidants [7]. Consequently, antioxidant capacity may be examined by a vast variety of assays based on different mechanisms such as hydrogen atom transfer, single electron transfer, reducing power, and metal chelation [9,10,11].

Research into the use of natural antioxidants as a replacement for synthetic antioxidants has been carried out since the 1970s [12]. Sage (*Salvia* spp. Labiatae) contains numerous phenolic compounds in the form of phenolic diterpenes (e.g., rosmanol, epirosmanol, isorosmanol, rosmadial, carnosic acid, and carnosol), phenolic acids (e.g., rosmarinic acid and simple phenolic acids) [8], and flavonoids and flavonoid-like compounds (e.g., luteolin 7-*O*-glucoside apigenin, hispidulin, and cirsimaritin) [13], all of which display antioxidant capacity [8]. For this reason, sage has regularly been employed as an antioxidant in meat and poultry products for several years [14,15,16,17,18,19].

In this study, the ability of sage (*Salvia* spp. Labiatae) extract (0.1% *w*/*w*) to reduce lipid oxidation in ground, uncured, cooked meat of porcine and bovine origin (60% pork, 40% beef) was examined. Specifically, the effectiveness of sage as an inhibitor of lipid oxidation measured as peroxide value (PV) and TBARS depending on genotype (five genotypes) and harvest time (three harvest times) of the sage plant was investigated. For determining the antioxidative capacity of the sage extract, some of the most commonly used methods were employed: radical scavenging activity via 2,2-diphenyl-1-picrylhydrazyl (DPPH), reducing power by the reduction of iron(III) to iron(II), and superoxide anion scavenging activity as well as determination of total phenolic content via the Folin–Ciocalteu method.

## 2. Materials and Methods

### 2.1. Sage Plant Material and Production of Sage Extracts

For this study, samples from the leaves of 15 different sage samples were used either as dried, milled sage or as ethanolic sage extracts. The sage plants (five genotypes, three different harvest times) were grown in the testing field of the University of Natural Resources and Applied Life Sciences Vienna in Großenzersdorf, Austria (48°12 N, 16°33 E) as described by Grausgruber-Gröger et al. [20] and collected during the summer of 2005. The sage species were identified by Prof. Johannes Novak from the Institute of Animal Nutrition and Functional Plant Compounds at the University of Veterinary Medicine Vienna, and the hebarium specimens are deposited at the institute. Table 1 shows an overview of the sage samples.

For production of sage extracts, leaves from the sage plants were dried and milled (Prochaska & Cie, Vienna, Austria). To 25 g of the dried sage sample, 200 mL ethanol (Merck, Darmstadt, Germany) was added, and the mixture was placed in a shaking water bath (1083, GFL, Hamburg, Germany) at 40 °C for 24 h. The extracts were collected in a round bottom flask and evaporated (rotary evaporator, R-144, Büchi, Flawil, Switzerland) at 50 °C. After evaporation, the dried extracts were dissolved in 25 mL ethanol. The concentrations of the ethanolic extracts are listed in Table A1.

### 2.2. Antioxidant Capacity and Total Phenolic Content

The antioxidant capacity of the sage extracts was analyzed according to three assays namely, radical scavenging activity, reducing power, and superoxide anion scavenging activity. Additionally, total phenolic content of the sage extracts was determined.

#### 2.2.1. Total Phenolic Content

Total phenolic content was measured by a modified version of the Folin–Ciocalteu-method [21] with a few additional modifications. The principle of this method is the reduction in the Folin–Ciocalteu reagent (phosphotungstic acid and phosphomolybdic acid) in basic medium by polyphenols from the sample, leading to a color change from yellow to blue. The sage extracts were diluted 1:10, 1:50, and 1:100 with distilled water, and 0.1 mL of each dilution mixed with 8.5 mL distilled water, and 0.5 mL Folin–Ciocalteu reagent (Sigma Aldrich, St. Louis, MO, USA). After 3–6 min, 1 mL 10% sodium carbonate (Merck, Darmstadt, Germany) solution was added, and the mixture then left to react in the dark for 1 h before measuring the color change photometrically (UV-120-02 Shimadzu, Kyoto, Japan) at 720 nm. Total phenolic content was calculated via a calibration curve of a catechin (Sigma Aldrich, St. Louis, MO, USA) serial dilution prepared in a similar way and reported as mg catechin equivalent per g extract yield (i.e., percentage in the ethanolic extract). 

#### 2.2.2. Radical Scavenging Activity

The radical scavenging activity was analyzed by a modified DPPH radical scavenging activity method by Hatano et al. [22] with the modifications described by Juntachote et al. [23]. The added violet DPPH radical reacts with the antioxidant to become discolored. A lighter color and a lower absorbance of the sample solution signify a faster radical reduction. The sage extracts were diluted to 200–900 mg/L with ethanol (Merck, Darmstadt, Germany). Duplicate dilutions 1:60, 1:30, 1:20, 1:15, 1:12, and 1:10 were prepared for each sage sample. To one preparation, 2.7 mL working solution (2.56 mg DPPH/100 mL; Honeywell-Fluka, Charlotte, NC, USA) was added; to the other, 2.7 mL methanol (blank) (Merck, Darmstadt, Germany). Photometric measurement (UV-120-02 Shimadzu, Kyoto, Japan) was done at 515 nm against methanol, and the absorbance of the blank was subtracted from the absorbance of the sample containing DPPH-solution. A diagram was created with the amount of unconverted DPPH (in percent) against the effective sample concentration (g sample/g DPPH) and thus, the concentration that is necessary to convert 50% of the DPPH (EC50) and, finally, the radical scavenging activity could be determined as radical scavenging activity = 1/EC50 (g DPPH/g sample).

#### 2.2.3. Reducing Power

The principle of the method according to Oyaizu [24] is the reduction of iron(III) to iron(II), which can be followed photometrically. The sage extracts were diluted with ethanol (Merck, Darmstadt, Germany), to a concentration of 600 mg/L, and their ability to reduce added iron(III) was analyzed (duplicate determinations) as described by Juntachote et al. [23] with a few modifications. The ethanolic extracts (0.5 mL) were mixed with 2.5 mL phosphate buffer (0.2 M, pH 6.6) and 2.5 mL potassium ferricyanide (1%) (both Honeywell-Riedel de Haën, Charlotte, NC, USA). After 20 min incubation at 50 °C, 2.5 mL 10% trichloroacetic acid (Roth, Karlsruhe, Germany) was added, and the mixture was then membrane filtered. The filtrate (2.5 mL) was mixed with an equal amount of distilled water and 0.5 mL of ferric chloride (1 g/L) (Merck, Darmstadt, Germany). Absorbance was measured photometrically at 700 nm (UV-120-02 Shimadzu, Kyoto, Japan), with a higher absorbance indicating a better reducing power.

#### 2.2.4. Superoxide Anion Scavenging Activity

Superoxide anion scavenging activity is the ability of an antioxidant to remove superoxide anion radicals. Superoxide anion scavenging activity was analyzed (duplicate determinations) according to the method described by Liu et al. [25] with the modifications described by Juntachote & Berghofer [26]. The principle of the method is that superoxide anions are formed in a non-enzymatic N-methylphenazonium methyl sulfate (PMS)-NADH system by oxidation of NADH and reduction of nitroblue tetrazolium (NBT). A total of 4 mg dried, milled sage was diluted in 2 mL tris buffer (Honeywell-Riedel de Haën, Charlotte, NC, USA) and 0.5 mL of this solution (or 0.5 mL tris buffer for the control/blank) was then mixed with 0.5 mL NBT (Sigma Aldrich, St. Louis, MO, USA), 0.5 mL NADH (Sigma Aldrich, St. Louis, MO, USA), and 0.5 mL PMS (Honeywell-Fluka, Charlotte, NC, USA) solutions. Absorbance was measured at 560 nm (UV-120-02 Shimadzu, Kyoto, Japan). The lower the absorbance, the higher the superoxide anion scavenging activity.

Relative superoxide anion scavenging activity (sample with the highest activity set to 100) was calculated as follows:

(1) $$\mathrm{Relative}\;\mathrm{superoxide}\;\mathrm{anion}\;\mathrm{scavenging}\;\mathrm{activity}=\left(1-\frac{\mathrm{abs}\left(\mathrm{sample}\;n\right)\;\mathrm{at}\;560\;\mathrm{nm}}{\mathrm{abs}\left(\mathrm{sample}\;\mathrm{with}\;\mathrm{lowest}\;\mathrm{abs}\right)\mathrm{at}\;560\;\mathrm{nm}}\right)*100+100$$

### 2.3. Meat Product Processing

Ground meat of pork (60%) and beef (40%), commonly sold as a mixed product in Austria, packaged in a modified gas atmosphere (73% O_2_, 22% CO_2_, 5% N_2_) was picked up on the day of production from the company Berger Ges.m.b.H & CoKG (Vienna, Austria) and was used on the same day for sample preparation. For sample preparation, 1.2 kg ground meat was mixed in a cutter with 2% iodized table salt and 0.1% (on a dry-weight basis) dried sage for the sage-containing samples and 2% iodized table salt for the control sample. Samples were pressed to 1 cm thickness, vacuum packaged, and heated in a water bath (Polystat cc1, Huber, Offenburg, Germany) for 1 h at 80 °C. The extensive cooking time was to induce heme iron release as well as to ensure inactivation of any vegetative microorganisms potentially present in the meat. After cooling, samples were homogenized in a cutter. For the storage trial, half of the samples were packaged in oxygen permeable cling film and stored in a fridge with a glass door at 7 °C for 14 days. The other half of the samples were used immediately for analysis of lipid oxidation (day 0).

### 2.4. Analysis of Lipid Oxidation

On day 0 (immediately after preparation) as well as on day 7 and day 14 of storage, lipid oxidation was measured as both PV and TBARS. PV quantifies hydroperoxides, which are primary lipid oxidation products, while TBARS is a measure of secondary lipid oxidation products [27].

#### 2.4.1. Peroxide Value (PV)

Firstly, fat was extracted from the meat sample by solubilization into n-hexane with subsequent filtration and evaporation. The extracted fat was frozen until the next day for analysis of PV. Five g of the extracted fat was weighed into a microbeaker followed by addition of 20 mL chloroform-pure acetic acid-mixture (Roth, Karlsruhe, Germany). PV determination was according to the method by Sully [28] as described in the DFG Unit method C-VI 6a [29], which is based on the principle that added potassium iodide (0.55 mL, 4.6 mol/L) (Roth, Karlsruhe, Germany) reacts with peroxide in the sample to form I_2_. The I_2_ is then determined by titration with sodium thiosulfate (0.01 mol/L), and with starch as the end-point indicator. The starch solution was prepared by mixing 1 g of soluble starch first with a small amount of cold, distilled water, and then with boiling water to a final volume of 200 mL. After cooling, the supernatant was used as the starch solution. Finally, PV can be calculated as

(2) $$PV=\frac{S\times C\times 1000}{m}$$

where *S* is mL sodium thiosulfate used in the titration, *C* is the concentration of sodium thiosulfate (0.01 mol/L), and m is the mass of the fat sample in g. Measurements were done in duplicate.

#### 2.4.2. Thiobarbituric Acid Active Substances (TBARS)

The method by Witte et al. [30] as modified by Piette & Raymond [31] was used for the analysis of TBARS. The method is based on the principle that carboxyl compounds in the sample react with thiobarbituric acid in acetate solution, hence, turning red, and can be determined photometrically at 530 nm. For the determination of TBARS, 20 g (day 0) or 10 g (day 7 and day 14) of sample material was homogenized (Ultra-Turrax T25, IKA™, Königswinter, Germany) in 50 mL 10% trichloroacetic acid (Roth, Karlsruhe, Germany), filled up to 100 mL, and filtered through a folded filter (MN 615 1/4). Five mL of the filtrate was heated with 5 mL TBA solution (5.73 g/L) (Sigma Aldrich, St. Louis, MO, USA) in a water bath (Polystat cc1, Huber, Offenburg, Germany) in boiling water for 5 min and then determined photometrically at 530 nm (UV-120-02, Shimadzu, Kyoto, Japan). The blank consisted of 5 mL TBA solution and 5 mL distilled water. TBARS, determined as mg MDA/kg meat sample, was calculated as

(3) $$C=\frac{A\times MW\times DF}{\varepsilon \times m}$$

where *C* is the concentration of MDA, *A* is the absorbance at 530 nm, *MW* is the molar weight of MDA (72.06 g/mol), *DF* is the dilution factor, *ε* is the extinction coefficient (1.35), and *m* is the weight of the sample. Results are the average of six measurements.

#### 2.4.3. Relative Prevention of Lipid Oxidation—Efficiency Factor

Relative prevention of lipid oxidation was calculated as an efficiency factor at days 7 and 14 of storage as PV or TBARS value of the control sample divided by the PV or TBARS value of the meat sample with added sage extract. The higher the efficiency factor, the better the ability of the sage extract to reduce lipid oxidation (PV or TBARS) in the meat sample.

### 2.5. Statistical Analysis

The Pearson correlation coefficients between the two measures for lipid oxidation (PV, TBARS) as pre-treated data in the form of the efficiency factors (see Section 2.4.3) and total phenolic content as well as the three measures for antioxidant capacity (DPPH radical scavenging activity, reducing power, and superoxide anion scavenging activity) were calculated using RStudio Version 2022.12.0 (Posit Software, Boston, MA, USA). *p* ≤ 0.05 was considered statistically significant.

Furthermore, the effect of storage time on PV and TBARS as well as the effect of harvest time and genotype, respectively, on PV and TBARS at days 0, 7, and 14 were analyzed (Microsoft Excel 2010) by applying an F-test to prove the similarity of variance followed by a *t*-test, where *p* ≤ 0.05 was considered statistically significant.

## 3. Results and Discussion

### 3.1. Antioxidant Capacity and Total Phenolic Content

Total phenolic content as well as antioxidant capacity in the form of radical scavenging activity, reducing power, and superoxide anion scavenging activity for each of the 15 sage samples are shown in Table 2. It is seen that the three different methods each find a different sage sample to be most efficient antioxidant with a fourth sample having the highest total phenolic content, confirming the fact that different methods for measuring antioxidant capacity yield different results.

Pearson correlation coefficients (Table 3) show that of the antioxidant capacity assays applied, superoxide anion scavenging activity correlated best with the measurements of lipid oxidation in the meat samples as a significant correlation between the superoxide anion scavenging ability and the level of PV at day 7 and TBARS at both day 7 and day 14 (correlation coefficients are negative because a lower abs signifies a better superoxide anion scavenging ability), while radical scavenging activity correlated to both PV and TBARS, but only at day 14, and there was no correlation for reducing power.

Based on the relative superoxide anion scavenging activity (highest scavenging activity set to base 100 assay, sage sample 9 has the strongest antioxidant capacity (Table 2). The superoxide anion (O_2_^−^) is known to relate to oxidation in meat via its formation during the oxidation of ferrous myoglobin (Mb) to the ferric metMb [32,33,34]. O_2_^−^ quickly yields hydrogen peroxide (H_2_O_2_), which is then free to react with metMb, forming prooxidative Mb species, which are able to initiate lipid oxidation [32]. *S. officinalis* has previously been shown to have a strong O_2_^−^ scavenging ability [35]. Flavonoids are acknowledged as efficient scavengers of O_2_^−^ [36], and *S. officinalis* L. is known to be rich in flavonoids [37].

It is clear from Table 2 that the correlation between total phenolic content and the various antioxidant capacity assays is not necessarily straightforward. However, total phenolic content does correlate to the reduction in lipid oxidation in the form of TBARS at day 7 (Table 3), and with P-values very close to being significant for PV at day 7 and TBARS at day 14 as well. That a high total phenolic content is not necessarily synonymous with a high antioxidant capacity is also evident from the literature. Some studies have found a good correlation between total phenolic content and antioxidant capacity as determined by various assays, e.g., in red, white, and rosé wines [38], in wild vegetables [39], in *S. officinalis* [40] of different origins [41], and for some Malvaceae family species but not for others [42]. On the other hand, one study [43] found no correlation between total phenolic content and antioxidant capacity of a different species of sage, *S. macrosiphon*, and another study [44] found that methanol/water extraction of *S. officinalis* resulted in the highest antioxidant capacity (including lowest TBARS), but the aqueous extract obtained by decoction resulted in the highest total phenolic content.

Evaluation of antioxidant capacity is usually performed using a model system, though this can only serve as a guideline [45], as the actual antioxidant capacity in a food will vary according to the physical location of the antioxidant within the food, the interaction of the antioxidant with other components of the food, and conditions such as heat treatment, etc. [46]. Thus, in addition to measuring antioxidant capacity in a model system, it is highly relevant to determine the effectiveness of the antioxidants in the food product in question [47]. When determining antioxidant capacity, it is recommended to use more than one method [11,48]. The selected assays should be able to provide antioxidative information that is directly related to the oxidative deterioration of the specific food product [10]. The choice of methods will depend on the antioxidative actions of the antioxidant [49,50], which for plant extracts are influenced by the solvent used as well as the extraction procedure employed [51]. For example, an assay involving redox reactions (transition metal chelation), an assay that works via hydrogen atom transfer, and an assay for scavenging relatively stable free radicals via electron transfer might be a suitable combination for determining the antioxidative capacity of a food product [48]. This highlights the importance of choosing an appropriate method for determination of antioxidant capacity depending on the nature of the food product, as also indicated by the results of the present study. For example, Fasseas et al. [52] measured TBARS and applied the DPPH assay as well as the crocin assay (radical scavenging activity) to determine the antioxidant capacity of sage and oregano essential oils in raw and cooked pork and beef. Their results showed a reduction in TBARS with addition of either essential oil, but these results did not correlate well with the results of neither the DPPH assay nor the crocin assay [52], indicating that a different method for determination of antioxidant activity could have provided a better correlation with TBARS. It should be noted that it is recommended to correlate the chosen chemical method for determination of lipid oxidation to a sensory test [27], although this was beyond the scope of the present study.

### 3.2. Lipid Oxidation in Meat Samples

After 0, 7, and 14 days of refrigerated storage, degree of lipid oxidation in the form of PV and TBARS varied according to the characteristics of the sage samples. From Table A2, it is seen that both PV and TBARS were reduced by the addition of any of the 15 samples of sage extract after 0, 7, and 14 days of storage, though to a varying degree. The reductions were to be expected, because sage has previously been proven to be an efficient inhibitor of lipid oxidation in meat and poultry [14,15,16,52,53,54]. The statistical analysis of the present results also showed an effect of storage time (*p* ≤ 0.05), lipid oxidation, unsurprisingly, increasing with increasing storage time (Table A2).

A previous study investigated bovine and porcine meat homogenized with 3% *w*/*w* sage (*Salvia officinalis* L.) essential oil and stored refrigerated (4 °C) in both the raw and the cooked stage for up to 12 days [52]. It was found that lipid oxidation (TBARS) was reduced in both types of meat throughout storage [52]. Two other studies [53,54] tested several natural antioxidants, including sage at a 0–1% *w*/*w* addition level. Sage was found to reduce lipid oxidation (TBARS) in patties made from either fresh pork or previously frozen pork during refrigerated storage (4 °C) in oxygen permeable cling film under retail conditions for nine days [53,54]. However, sage did not have a significant effect on TBARS in cooked patties [53]. 

In a different study, mechanically separated chicken meat was mixed with sage (*S. officinalis* L.) in the form of either a water extract, an ethanol extract, or an essential oil [14]. After frozen storage (−18 °C) in a vacuum (90% evacuation of air) for up to nine months, lipid oxidation (TBARS) was measured. The authors found that both the water extract of sage and the essential oil (40% and 70% vol/vol) significantly reduced the degree of lipid oxidation in the mechanically separated, frozen chicken compared to the control sample without added sage [14]. In another study, the effect of addition of 0.10% sage (dry plant) to cooked chicken breast meatballs was investigated. Lipid oxidation was measured as headspace hexanal after up to 144 h (six days) of frozen storage (−20 °C) [15]. Sage was found to be effective in reducing the amount of headspace hexanal [15], which is an important secondary lipid oxidation product [55] and, therefore, a measure of the extent of lipid oxidation.

**Figure 1:**
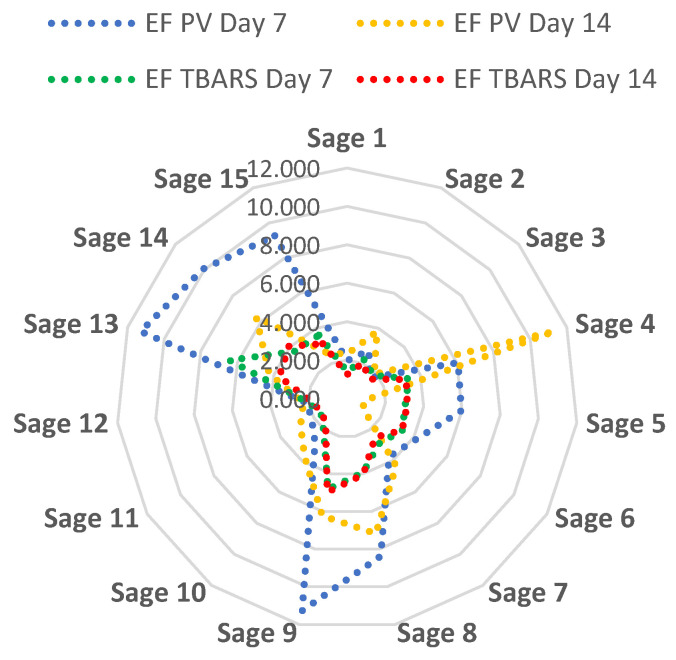
Relative prevention of lipid oxidation expressed as the efficiency factor (EF) for each sage extract sample at day 7 and day 14 of refrigerated storage calculated as the ability of the sage extract to inhibit lipid oxidation measured as peroxide value (PV) and thiobarbituric acid reactive substances (TBARS) in an uncured, cooked meat sample. The higher the efficiency factor, the better the ability of the sage extract to reduce lipid oxidation (PV or TBARS) in the meat sample.

Figure 1 illustrates the efficiency factor for each of the 15 sage extracts in relation to both PV and TBARS measurements on days 7 and 14 of refrigerated storage. On day seven of storage, meat containing sage sample 13 showed the highest efficiency factor (lowest degree of lipid oxidation compared to the corresponding control sample), followed by sample 9 for both PV- and TBARS-measurements. On day 14, meat containing sage sample 4 (PV) and sage sample 9 (TBARS), respectively, showed the best prevention of lipid oxidation (highest efficiency factor). Overall, extracts of sage samples 9 and 14 were in the top four for prevention of lipid oxidation for both PV and TBARS on both days of storage.

Genotype is known to influence antioxidant potential of sage [20,56]. In the present study, the best-performing sage extract samples were of the *S. officinalis* L. accession ‘Foggia’, IT (sage 4, 9, and 14) and *S. officinalis* L. of the breeding line ‘AT F1 01 24′ (sage 13 and 8) (Table 1). The statistical analysis confirms that *S. officinalis* L. accession ‘Foggia’ generally performed better than the other genotypes in reducing lipid oxidation in this ground, cooked meat product. At day 7, there was a statistically significant difference (*p* ≤ 0.05) between *S. officinalis* accession from Foggia, IT and both *S. lavandulifolia* cv. ‘Grete Stölzle’ and *S. officinalis* AT F1 01 11 based on PV, and between *S. officinalis* accession from Foggia, IT and *S. officinalis* AT F1 01 11 based on TBARS. At day 14, there was a significant difference (*p* ≤ 0.05) between *S. officinalis* accession from Foggia, IT and *S. lavandulifolia* cv. ‘Grete Stölzle’, *S. officinalis* AT F1 01 11, and *S. officinalis* AT F1 01 24 based on PV, and between *S. officinalis* accession from Foggia, IT and *S. officinalis* AT F1 01 11 based on TBARS.

Harvest time has been shown to affect the antioxidant capacity of sage [56,57], though the effect on lipid oxidation does not seem to be clear in our case with samples harvested throughout the summer performing well. The only statistically significant differences were found for PV at day 0 (between all three months), and between July and August for TBARS at day 0 (*p* ≤ 0.05). Hence, genotype seems to be more important than harvest time for ability to reduce lipid oxidation in a ground, uncured, cooked meat sample, though harvest times throughout spring and summer should be investigated in future studies, which should also include additional *S.* spp. Labiatae genotypes. It might also be beneficial to investigate the use of different extraction conditions, as extraction procedure and solvent have been found to influence the antioxidative capacity of sage [14,51].

## 4. Conclusions

All 15 sage extract samples were able to reduce lipid oxidation in ground, uncured, cooked porcine and bovine meat (60%/40% mixture) as determined by PV and TBARS measurements. Nonetheless, genotype and harvest time of the sage plant both influenced the antioxidant capacity of the resultant extract, with genotype, by far, being the most important factor. In this case, extracts of *S. officinalis* accession from Foggia, Italy, performed best when looking at the entire 14-day storage period and considering both PV and TBARS measurements. In the future, it could be of interest to systematically study even more *S.* spp. Labiatae genotypes in connection with different harvest times throughout spring and summer as well as various extraction procedures and solvents to establish the most efficient combination for the use of sage as an antioxidant in meat and meat products.

Furthermore, the results highlighted that care must be taken when choosing one or more methods for determination of antioxidant capacity in a model system. Of the limited number of methods evaluated in this study, the best correlation to inhibition of lipid oxidation in the meat product was determination of superoxide anion scavenging activity in the sage extract.

## Figures and Tables

**Table 1:** Harvest time and genotype of the 15 sage samples investigated for antioxidant capacity.

Sage	Harvest Time	Genotype
Sage 1	Primo June	*S. lavandulifolia* cv. ‘Grete Stölzle’
Sage 2	Primo June	*S. officinalis* AT F1 01 11
Sage 3	Primo June	*S. officinalis* AT F1 01 24
Sage 4	Primo June	*S. officinalis* accession from Foggia, IT
Sage 5	Primo June	*S. officinalis* R1
Sage 6	Primo July	*S. lavandulifolia* cv. ‘Grete Stölzle’
Sage 7	Primo July	*S. officinalis* AT F1 01 11
Sage 8	Primo July	*S. officinalis* AT F1 01 24
Sage 9	Primo July	*S. officinalis* accession from Foggia, IT
Sage 10	Primo July	*S. officinalis* R1
Sage 11	Primo August	*S. lavandulifolia* cv. ‘Grete Stölzle’
Sage 12	Primo August	*S. officinalis* AT F1 01 11
Sage 13	Primo August	*S. officinalis* AT F1 01 24
Sage 14	Primo August	*S. officinalis* accession from Foggia, IT
Sage 15	Primo August	*S. officinalis* R1

**Table 2:** Total phenolic content and antioxidant capacity of 15 sage samples were determined as relative radical scavenging activity, reducing power, and superoxide anion scavenging activity. The sample with the highest total phenolic content and highest antioxidant capacity, respectively, according to each method, is marked in bold.

	Total Phenolic Content (mg Catechin Equivalent/g Extract Yield)	Radical Scavenging Activity (1/EC50)	Reducing Power (abs)	Superoxide Anion Scavenging Activity
Sage 1	133	1.185	**0.566**	42
Sage 2	78	1.420	0.375	58
Sage 3	133	1.274	0.496	76
Sage 4	134	2.141	0.484	61
Sage 5	110	1.992	0.457	59
Sage 6	112	1.595	0.350	47
Sage 7	99	2.012	0.436	57
Sage 8	100	1.764	0.394	81
Sage 9	145	1.757	0.448	**100**
Sage 10	131	1.582	0.447	72
Sage 11	87	1.449	0.322	41
Sage 12	**174**	1.477	0.341	43
Sage 13	162	1.414	0.461	85
Sage 14	111	**2.364**	0.491	71
Sage 15	102	1.443	0.520	81

**Table 3:** Correlation between lipid oxidation day 7 and day 14 measured as peroxide value (PV) and thiobarbituric acid reactive substances (TBARS) and total phenolic content and antioxidant capacity of sage measured as, radical scavenging activity determined via a modified 2,2-diphenyl-1-picrylhydrazyl (DPPH) method, reducing power, and superoxide anion scavenging activity, respectively; n = 15. Significant correlations in bold.

		Total Phenolic Content		Radical Scavenging Activity		Reducing Power		Superoxide Anion Scavenging Activity	
		Day 7	Day 14	Day 7	Day 14	Day 7	Day 14	Day 7	Day 14
PV	Pearson corr.	0.496	0.308	0.416	**0.573**	0.273	0.155	**−0.765**	−0.364
	*p*-value	0.060	0.265	0.123	0.026	0.325	0.581	0.001	0.183
TBARS	Pearson corr.	**0.585**	0.469	0.284	**0.574**	0.141	0.075	**−0.683**	**−0.685**
	*p*-value	0.022	0.078	0.305	0.025	0.617	0.789	0.005	0.005

## Data Availability

The data presented in the study are available from the corresponding author upon request.

## Appendix A

**Table A1:** Final concentration (g/L) in the ethanolic sage extracts.

Sage	Extract Concentration (g/L)
Sage 1	74.0
Sage 2	96.4
Sage 3	98.0
Sage 4	85.2
Sage 5	72.0
Sage 6	2.8
Sage 7	87.2
Sage 8	152.8
Sage 9	122.8
Sage 10	84.0
Sage 11	83.6
Sage 12	80.8
Sage 13	108.4
Sage 14	79.6
Sage 15	94.4

**Table A2:** Peroxide value (PV) (active O_2_ in 1/8 mmol/kg meat) and thiobarbituric acid reactive substances (TBARS) (mg malondialdehyde/kg meat) after 0, 7, and 14 days of refrigerated storage for cooked beef/pork samples with added sage. The sage sample with the lowest PV and TBARS, respectively, at each time point is marked in bold.

	PV	Day 0	Day 7	Day 14	TBARS	Day 0	Day 7	Day 14
Control 1–3		1.154	12.211	25.154		0.232	5.284	6.202
Sage 1		1.092	6.203	10.870		0.112	3.747	4.946
Sage 2		1.104	4.453	6.580		0.085	2.359	3.056
Sage 3		0.932	7.483	13.324		0.085	2.909	4.075
Control 4–6		0.716	11.183	21.221		0.405	4.523	5.975
Sage 4		0.524	1.845	**1.875**		0.155	1.343	1.847
Sage 5		0.587	1.874	2.322		0.157	1.487	1.903
Sage 6		0.501	2.674	2.242		0.165	1.366	1.815
Control 7–9		0.488	7.427	14.101		0.416	4.594	5.676
Sage 7		0.436	1.997	3.254		**0.078**	1.605	2.291
Sage 8		0.341	0.887	1.968		0.088	1.167	1.409
Sage 9		0.320	**0.659**	2.265		0.129	0.956	**1.143**
Control 10–12		0.527	10.302	20.664		0.434	4.538	5.867
Sage 10		0.407	3.631	5.506		0.149	2.195	3.085
Sage 11		**0.244**	4.835	7.370		0.142	2.885	3.991
Sage 12		0.413	4.894	9.017		0.139	2.239	3.191
Control 13–15		0.611	10.16	16.850		0.533	5.219	6.154
Sage 13		0.420	0.899	3.955		0.223	**0.811**	1.655
Sage 14		0.399	1.009	2.646		0.209	1.417	1.512
Sage 15		0.384	1.096	6.453		0.228	1.443	1.966
